# The Anti-Tumor Effect of A_3_ Adenosine Receptors Is Potentiated by Pulsed Electromagnetic Fields in Cultured Neural Cancer Cells

**DOI:** 10.1371/journal.pone.0039317

**Published:** 2012-06-25

**Authors:** Fabrizio Vincenzi, Martina Targa, Carmen Corciulo, Stefania Gessi, Stefania Merighi, Stefania Setti, Ruggero Cadossi, Pier Andrea Borea, Katia Varani

**Affiliations:** 1 Department of Clinical and Experimental Medicine, Pharmacology Unit, University of Ferrara, Ferrara, Italy; 2 Igea Biophysics Laboratory, Carpi, Italy; University of South Alabama, United States of America

## Abstract

A_3_ adenosine receptors (ARs) play a pivotal role in the development of cancer and their activation is involved in the inhibition of tumor growth. The effects of pulsed electromagnetic fields (PEMFs) on cancer have been controversially discussed and the detailed mechanisms are not yet fully understood. In the past we have demonstrated that PEMFs increased A_2A_ and A_3_AR density and functionality in human neutrophils, human and bovine synoviocytes, and bovine chondrocytes. In the same cells, PEMF exposure increased the anti-inflammatory effect mediated by A_2A_ and/or A_3_ARs. The primary aim of the present study was to evaluate if PEMF exposure potentiated the anti-tumor effect of A_3_ARs in PC12 rat adrenal pheochromocytoma and U87MG human glioblastoma cell lines in comparison with rat cortical neurons. Saturation binding assays and mRNA analysis revealed that PEMF exposure up-regulated A_2A_ and A_3_ARs that are well coupled to adenylate cyclase activity and cAMP production. The activation of A_2A_ and A_3_ARs resulted in the decrease of nuclear factor-kappa B (NF-kB) levels in tumor cells, whilst only A_3_ARs are involved in the increase of p53 expression. A_3_AR stimulation mediated an inhibition of tumor cell proliferation evaluated by thymidine incorporation. An increase of cytotoxicity by lactate dehydrogenase (LDH) release and apoptosis by caspase-3 activation in PC12 and U87MG cells, but not in cortical neurons, was observed following A_3_AR activation. The effect of the A_3_AR agonist in tumor cells was enhanced in the presence of PEMFs and blocked by using a well-known selective antagonist. Together these results demonstrated that PEMF exposure significantly increases the anti-tumor effect modulated by A_3_ARs.

## Introduction

Increasing evidence demonstrates that adenosine affects numerous pathophysiological processes including the regulation of cell death and proliferation [1], [2]. Adenosine interacts with four G-protein coupled receptors named as A_1_, A_2A_, A_2B_ and A_3_ adenosine receptors (ARs). A_1_ and A_3_ARs inhibit adenylate cyclase activity and decrease cAMP production whilst A_2A_ and A_2B_ARs exert an increase of cAMP accumulation [3]. The A_3_ARs have been involved in the regulation of the cell cycle and both pro- and antiapoptotic effects are closely associated with the level of receptor activation [4]. A_3_ARs are involved in the modulation of mitogen-activated protein kinase (MAPK) activity and in the regulation of extracellular signal-regulated kinases (ERK1/2) [5]. It has been accepted that A_3_ARs are highly expressed in tumor cells showing an important role in the development of cancer [6]–[10]. The tumor cell growth inhibition was found in different models as rat Nb2-11C and mouse Yac-1 lymphoma, B16-F10 melanoma, MCA sarcoma, PC3 prostate carcinoma, MIA-PaCa pancreatic carcinoma, Hep-3B hepatocellular carcinoma and HCT-116 colon carcinoma cells [11]–[14]. From the cellular point of view, the A_3_AR agonist 2-chloro-*N* ^6^-(3-iodobenzyl)adenosine-5′-*N*-methyl-uronamide (Cl-IB-MECA) inhibited tumor growth via de-regulation of the NF-kB signal transduction pathways, resulting in apoptosis of tumor cells [10]. Pharmacological studies demonstrated that A_3_AR agonists inhibited the growth of melanoma cells, promoted the proliferation of bone marrow cells, reduced cell viability in human breast cancer cells and induced arrest of cell cycle progression in human lung cancer cells [15], [16]. Moreover Cl-IB-MECA enhanced apoptosis via the modulation of nuclear factor-kappa B (NF-kB) signaling pathway in thyroid cancer cells and reduced the ability of prostate cancer cells to migrate *in vitro* and metastasize *in vivo* [17], [18]. In addition, preclinical and Phase I studies showed that A_3_AR agonists are safe and well tolerated in humans and thus may be considered possible therapeutic agents for certain cancer diseases [5].

Large research activity has raised around the mechanisms of interaction between low frequency magnetic fields and biological systems [19]. Different physiological systems seem to be influenced by electromagnetic field (EMF) exposure as revealed from *in vitro* experiments in various cells or tissues [20]–[25]. Recently, it has been reported a correlation between EMF exposure and neurodegenerative diseases as Alzheimer or Parkinson diseases [26]–[28]. In addition, pulsed electromagnetic fields (PEMFs) therapy significantly reduced post-operative pain and narcotic use in the immediate post-operative period by a mechanism that involve endogenous interleukin-1β (IL-1β) in the wound bed [29]. While some researchers associate EMF exposure with carcinogenesis [30], [31], other studies of experimental models and human cancers have shown that EMF does not increase the risk of several cancer types, and that treatment with tumor-specific frequencies is feasible and well tolerated and may have biological efficacy in patients with advanced tumors [32]–[34]. Moreover, the exposure of female C3H/HeJ mice bearing mammary adenocarcinoma to a frequency of 120 Hz at intensities of 4 and 5 mT resulted in a significant reduction in the tumor growth, which is a phenomenon associated with angiogenesis inhibition [35]. The exposure of female athymic nude mice with human breast cancer xenografts to a frequency of 120 Hz with an intensity of 15 mT, either alone or in combination with gamma radiation, resulted in decreased growth and reduced vascularization of the tumors [36]. Similarly, the effect of 50 Hz at 0.5 µT and 0.5 mT on the development of chemically induced foci in rat livers showed a slight inhibition of their formation [37]. In a recent work, the application of EMF inhibits preneoplastic lesions chemically induced in the rat liver through the reduction of cell proliferation, without altering the apoptosis process [38]. Novel findings have demonstrate that the magnetic field combined with X-Ray mediate a survival improvement and tumor inhibition in hepatoma-implanted mice [39]. In multidrug resistance (MDR) osteosarcoma cell line, PEMFs increased doxorubicin binding ability to DNA and inhibited cell growth, suggesting that PEMFs may be useful as a local treatment for MDR osteosarcoma [40].

In the current study we investigated whether PEMFs modulate the expression and the effect of A_3_ARs in different cells represented by rat adrenal pheochromocytoma cells (PC12) and human glioblastoma cell lines (U87MG) in comparison with rat cortical neurons. Using these cellular models we highlighted the effect of PEMFs on A_3_AR stimulation in NF-kB and p53 activation, cell proliferation, cytotoxicity and apoptosis. These results indicate the possibility that the anti-tumor effect mediated by A_3_ARs could be potentiated by a non invasive stimulus represented by PEMFs.

## Results

### PEMFs up-regulated A_2A_ and A_3_AR Density and Expression

The first objective of the current study was to verify if affinity and density of A_1_, A_2A_, A_2B_ and A_3_ARs were affected by PEMFs in rat cortical neurons, PC 12 and U87MG cells. In particular, Table 1 reports the affinity (K_D_) and density (Bmax) values for ARs in membranes or intact cells derived from rat cortical neurons, U87MG cells and untreated or NGF-treated PC12 cells in the absence and in the presence of PEMFs at 1.5 mT. Affinity values of ARs were not affected by PEMF treatment in the cells examined whilst A_2A_ and A_3_AR density was significantly increased.

The PEMF treatment of primary culture of rat cortical neurons at 1.5 mT induced an up-regulation of A_2A_AR density respect to control conditions in both preparation such as membranes or intact cells (Table 1). The exposure with PEMFs of untreated or NGF-treated PC12 cells induced an up-regulation of A_2A_AR density with a 2.20 or 2.10 fold of increase versus controls in membrane preparation, respectively. The A_2A_AR up-regulation was also evident on membranes from U87MG cells where Bmax values increased from 91±9 (control condition) to 207±19 (PEMFs treatment) fmol/mg protein. Similar results on A_2A_AR up-regulation were also obtained performing the saturation binding experiments with intact cells (Table 1).

**Table 1:** Affinity and density of A_1_, A_2A_, A_2B_ and A_3_ARs in rat cortical neurons, untreated or NGF-treated PC12 cells and U87MG membranes or cells in the absence or in the presence of PEMFs.

	A_1_ARs		A_2A_ARs		A_2B_ARs		A_3_ARs	
	K_D_	Bmax	K_D_	Bmax	K_D_	Bmax	K_D_	Bmax
**Rat Neurons** a	0.78±0.06	180±20	0.74±0.05	50±4	1.52±0.11	32±3	2.11±0.14	36±3
**+ PEMFs**	0.73±0.05	178±19	0.79±0.06	104±8*	1.57±0.12	30±3	2.14±0.20	76±7*
**PC12** a	1.12±0.10	46±4	1.71±0.09	152±12	1.72±0.16	65±6	2.05±0.18	94±9
**+ PEMFs**	1.17±0.11	45±4	1.85±0.14	335±27*	1.77±0.15	62±6	2.18±0.16	215±20*
**NGF-PC12** a	1.11±0.09	42±4	1.65±0.15	164±14	1.73±0.15	63±5	2.21±0.15	102±9
**+ PEMFs**	1.13±0.08	44±4	1.72±0.06	345±32*	1.76±0.16	64±4	2.38±0.16	222±21*
**U87MG** a	1.01±0.08	28±2	1.58±0.13	91±9	1.81±0.17	85±9	2.14±0.22	93±8
**+ PEMFs**	1.03±0.09	30±2	1.64±0.14	207±19*	1.83±0.16	88±9	2.01±0.19	216±18*
**Rat Neurons** b	0.75±0.06	7.3±0.7	0.72±0.06	2.4±0.2	1.61±0.14	1.4±0.1	2.02±0.17	1.7±0.1
**+ PEMFs**	0.79±0.07	7.6±0.6	0.81±0.07	4.6±0.5*	1.68±0.11	1.2±0.1	2.11±0.19	3.1±0.3*
**PC12** b	1.08±0.09	2.3±0.2	1.77±0.11	7.6±0.5	1.75±0.12	2.7±0.2	2.01±0.15	3.4±0.2
**+ PEMFs**	1.14±0.10	2.2±0.2	1.89±0.13	17.1±1.2*	1.81±0.10	2.5±0.2	2.13±0.14	8.4±0.6*
**NGF-PC12** b	1.18±0.11	1.9±0.2	1.71±0.14	8.4±0.7	1.79±0.13	2.6±0.2	2.19±0.13	3.7±0.4
**+ PEMFs**	1.17±0.10	2.0±0.2	1.69±0.10	19.4±2.1*	1.84±0.15	2.4±0.2	2.24±0.15	8.6±0.7*
**U87MG** b	1.07±0.09	1.5±0.1	1.64±0.11	2.9±0.3	1.85±0.14	2.8±0.2	2.09±0.18	3.2±0.3
**+ PEMFs**	1.11±0.10	1.6±0.1	1.72±0.15	8.6±0.9*	1.78±0.13	2.9±0.2	2.05±0.17	8.3±0.8*

a membranes;

b intact cells. Affinity and density are expressed as K_D_, nM and Bmax, fmol/mg protein, respectively.

[^3^H]-DPCPX saturation binding for A_1_ARs, [^3^H]-ZM 241385 saturation binding for A_2A_ARs, [^3^H]-MRE2029F20 saturation binding for A_2B_ARs, [^125^I]AB-MECA saturation binding for A_3_ARs. Data are expressed as mean (n = 6) ± SEM. Differences were considered significant at a value of p<0.01 vs the examined cells in the absence of PEMFs (*).

PEMF treatment induced an up-regulation of A_3_ARs by 2.11 or 2.32 fold on rat cortical neurons and on U87MG cells respect to control condition in membrane preparation, respectively (Table 1). Similar effects were obtained exposing untreated or NGF-treated PC12 cells with PEMFs at 1.5 mT. A_3_AR Bmax values increased from 94±9 (control condition) to 215±20 (PEMF treatment) fmol/mg protein in membrane from untreated PC12 cells. In membrane preparation from NGF-treated PC12 cells A_3_AR density increased from 102±9 (control condition) to 222±21 (PEMFs treatment) fmol/mg protein. Similarly, the A_3_AR up-regulation were also observed performing the saturation binding experiments with intact cells (Table 1).

**Figure 1:**
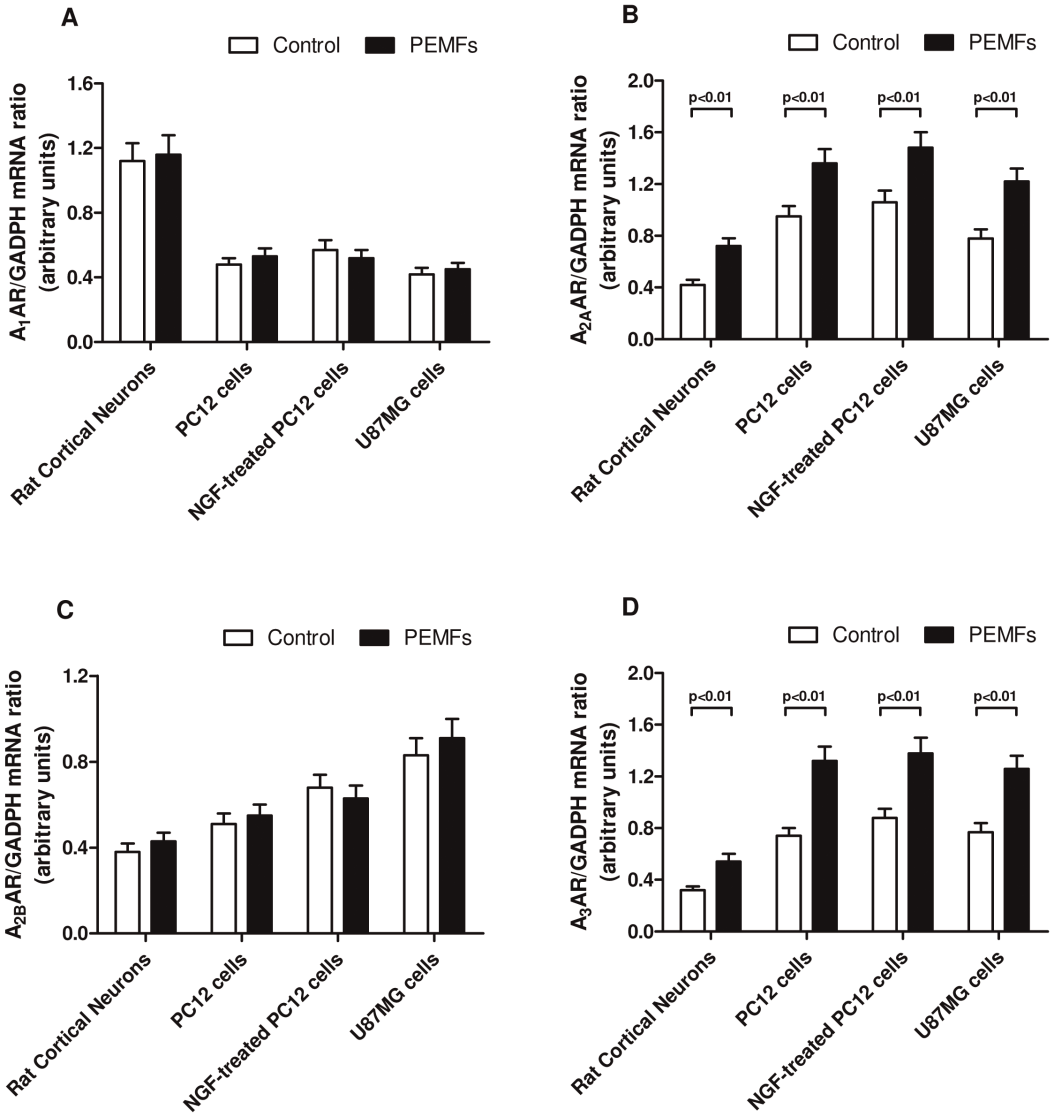
PEMFs increased A_2A_ and A_3_AR mRNA levels. Relative mRNA expression of A_1_, A_2A_, A_2B_ and A_3_ARs in rat cortical neurons, untreated or NGF-treated PC12 cells and U87MG cells in the absence or in the presence of PEMFs. Values are the mean (n = 6) ± SEM.

Figure 1 shows the mRNA relative expression of A_1_, A_2A_, A_2B_ and A_3_ARs in rat cortical neurons, untreated or NGF-treated PC12 cells and U87MG cells in the absence and in the presence of PEMFs. Interestingly A_2A_ and A_3_AR mRNA levels were up-regulated after PEMF treatment confirming the increase of their density found in saturation binding experiments (Table 1).

### A_2A_ and A_3_AR Stimulation and PEMFs Mediated a Significant Modulation in cAMP Production

**Figure 2:**
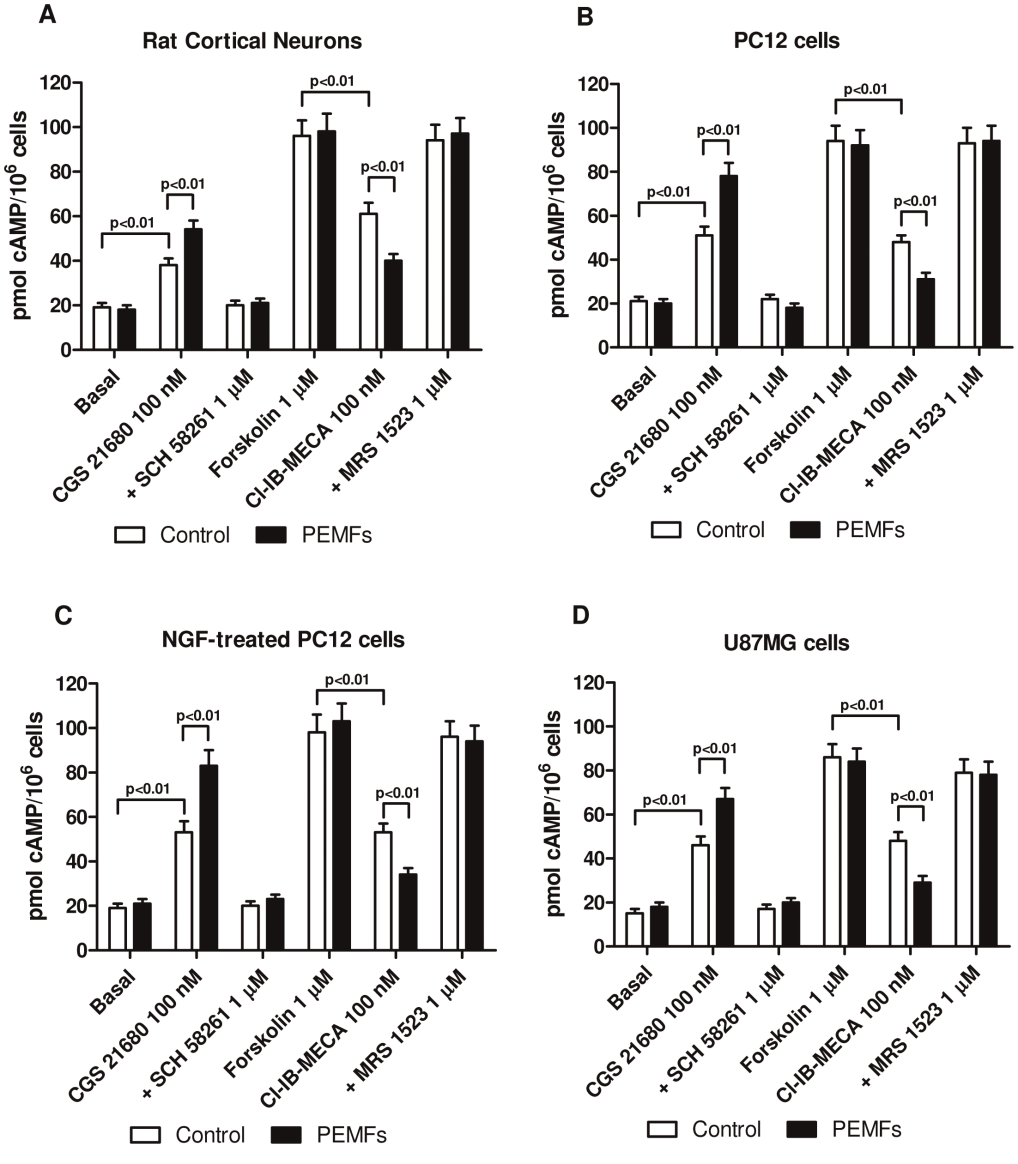
cAMP modulation by PEMFs up-regulated A_2A_ and A_3_ARs. Effect of a well-known A_2A_AR agonist and antagonist (CGS 21680, 100 nM; SCH 58261, 1 µM) or A_3_AR agonist and antagonist (Cl-IB-MECA, 100 nM; MRS 1523, 1 µM) in rat cortical neurons, untreated or NGF-treated PC12 cells and U87MG cells in the absence or in the presence of PEMFs on cAMP production. Values are the mean (n = 6) ± SEM.

The A_2A_ARs are coupled to stimulation of adenylate cyclase via Gs proteins which mediates an increase of cAMP production. In rat cortical neurons, U87MG cells and untreated or NGF-treated PC12 cells, CGS 21680 at the 100 nM concentration was able to mediate a significant increase in cAMP formation that is potentiated in the cells treated with PEMFs. In all the experimental conditions examined, SCH 58261 at the 1 µM concentration was able to completely block the effect of CGS 21680. The effect of the A_3_AR agonist Cl-IB-MECA (100 nM) was evaluated in the presence of 1 µM forskolin and 0.5 mM Ro 20-1724. This experimental condition was chosen for the low basal levels (15–20 pmol cAMP per assay) which hamper the evaluation of a direct inhibitory effect. Cl-IB-MECA mediated a significant decrease of cAMP production and this effect was significantly amplified in the cells treated with PEMFs (Fig. 2). Moreover, in all the cells examined, the presence of MRS 1523 (1 µM) inhibited the effect of Cl-IB-MECA confirming the involvement of A_3_ARs in the modulation of cAMP production.

### A_2A_ and A_3_AR Stimulation Mediated a Significant Inhibition of NF-kB Activation in the Presence of PEMFs

**Figure 3:**
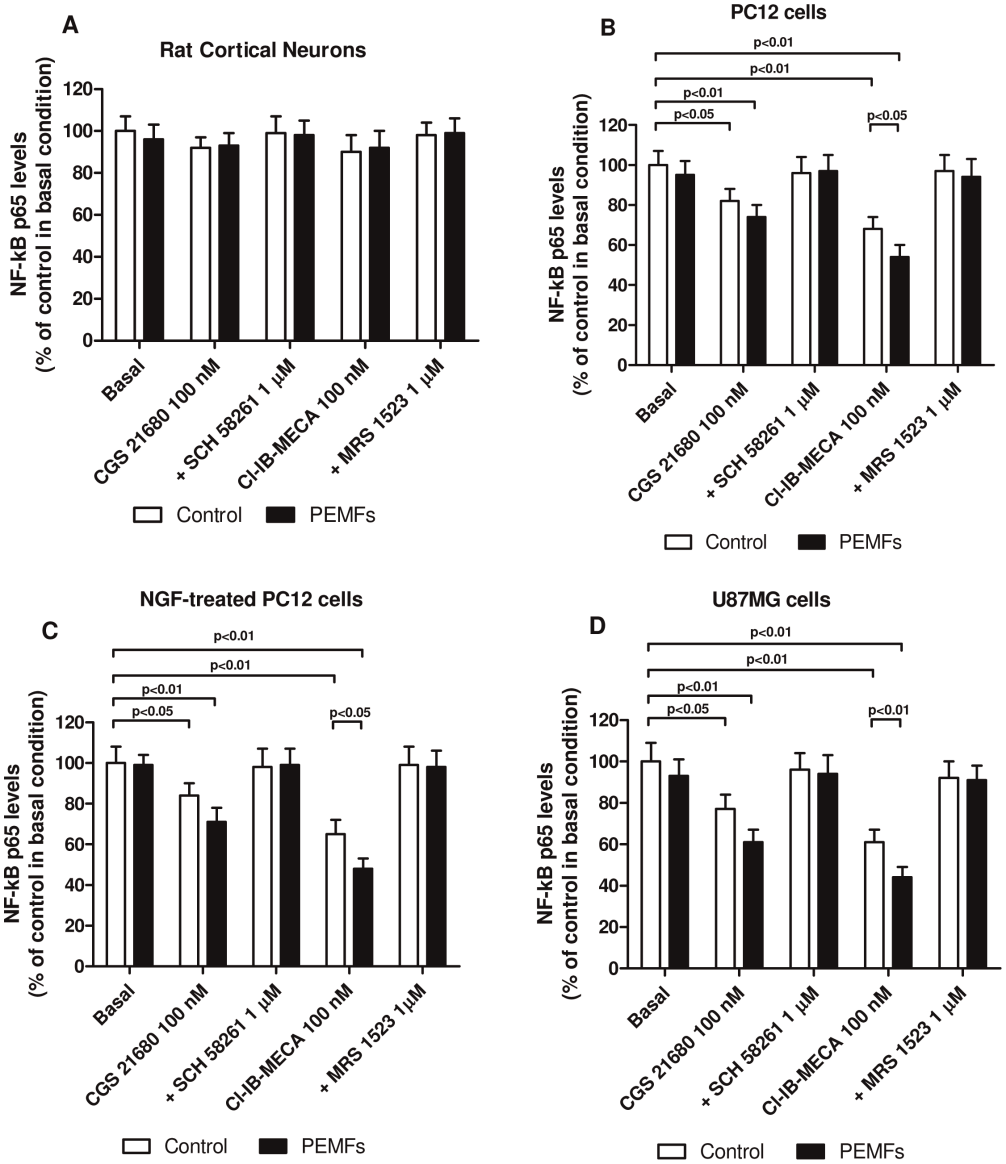
Inhibition of NF-kB activation by the co-presence of PEMFs and A_2A_ or A_3_AR agonists. Effect of a well-known A_2A_AR agonist and antagonist (CGS 21680, 100 nM; SCH 58261, 1 µM) or A_3_AR agonist and antagonist (Cl-IB-MECA, 100 nM; MRS 1523, 1 µM) in rat cortical neurons, untreated or NGF-treated PC12 cells and U87MG cells in the absence or in the presence of PEMFs on NF-kB activation which was evaluated by detecting phosphorylated p65 proteins in nuclear extracts. Values are the mean (n = 6) ± SEM.

The effect of A_2A_ or A_3_AR stimulation on NF-kB p65 subunit activation was investigated in untreated or NGF-treated PC12 cells and U87MG cells respect to rat cortical neurons revealing the capability of the A_2A_ (CGS 21680) or A_3_AR (Cl-IB-MECA) agonists to significantly decrease the activation of NF-kB in the tumor cells after 24 hours of treatment (Fig. 3). PEMF exposure was able to enhance the A_2A_ or A_3_AR agonist-induced reduction of NF-kB activation. The A_3_AR stimulation mediated a more evident inhibition of NF-kB than A_2A_ activation both in the presence or in the absence of PEMFs. Interestingly, the simultaneous treatment with A_3_AR agonist and PEMFs mediated a significant inhibition of NF-kB levels respect to the A_3_AR activation alone. The effect of these agonists was blocked by the use of well known A_2A_ or A_3_AR antagonists such as SCH 58261 and MRS 1523, respectively (Fig. 3).

### Activation of A_3_AR in the Presence of PEMFs Increased p53 levels Leading to Inhibition of Tumor Cell Proliferation

**Figure 4:**
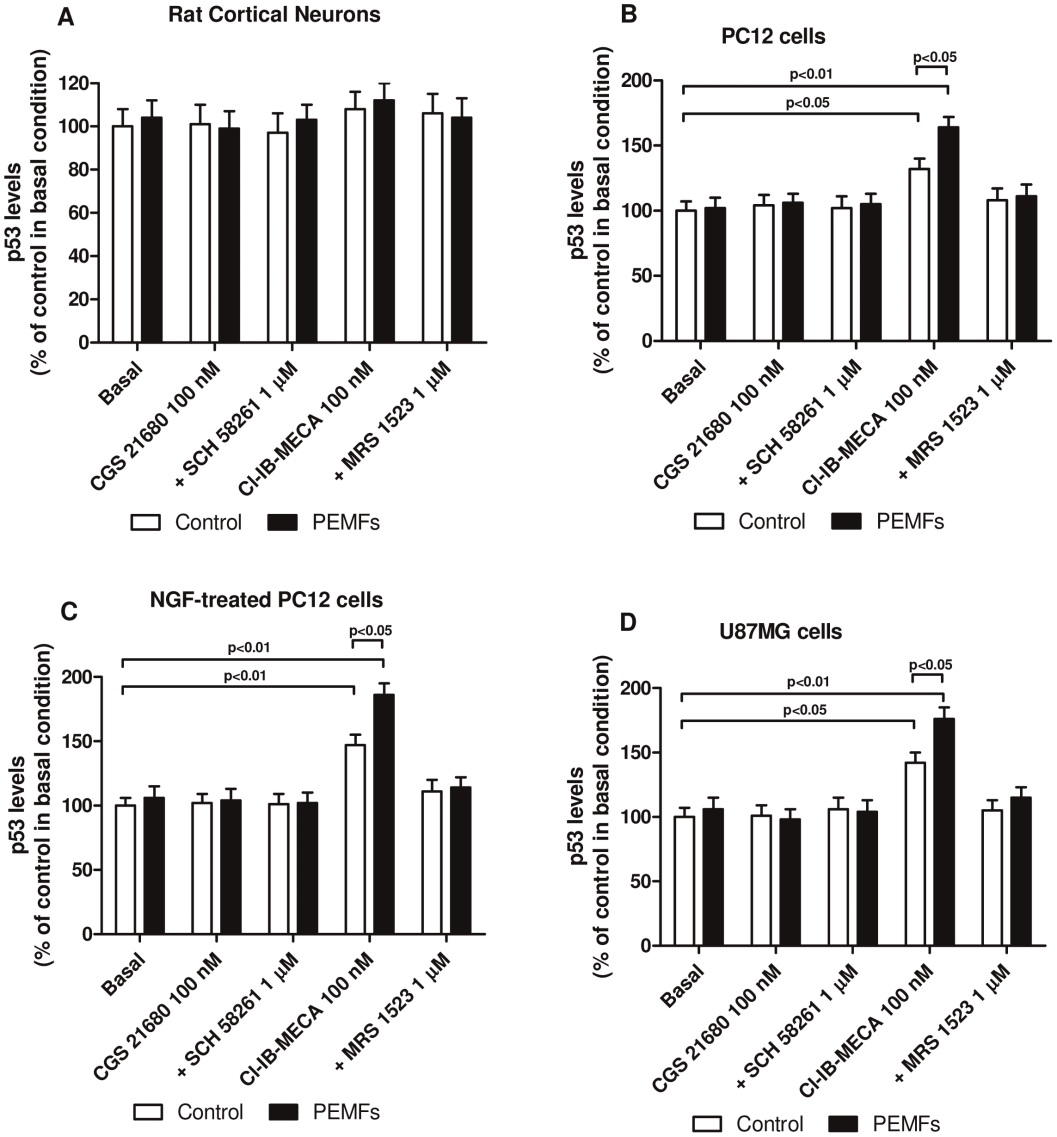
A_3_AR agonist and PEMFs increased p53 levels. Effect of a well-known A_2A_AR agonist and antagonist (CGS 21680, 100 nM; SCH 58261, 1 µM) or A_3_AR agonist and antagonist (Cl-IB-MECA, 100 nM; MRS 1523, 1 µM) in rat cortical neurons, untreated or NGF-treated PC12 cells and U87MG cells in the absence or in the presence of PEMFs on p53 levels. Values are the mean (n = 6) ± SEM.

The A_2A_AR agonist CGS 21680 was not able to modulate p53 protein levels neither in the absence nor in the presence of PEMFs in the different cell types examined. Conversely, the A_3_AR agonist Cl-IB-MECA (100 nM) mediated a significant increase of p53 expression in the tumor cell lines examined without affecting the p53 levels of the rat control cortical neurons (Fig. 4). Although PEMF exposure alone was not able to modify p53 expression, the simultaneous treatment of tumor cells with PEMFs and Cl-IB-MECA resulted in a further significant increase of p53 protein levels in comparison with Cl-IB-MECA alone (p<0.05, Fig. 4B-D).

**Figure 5:**
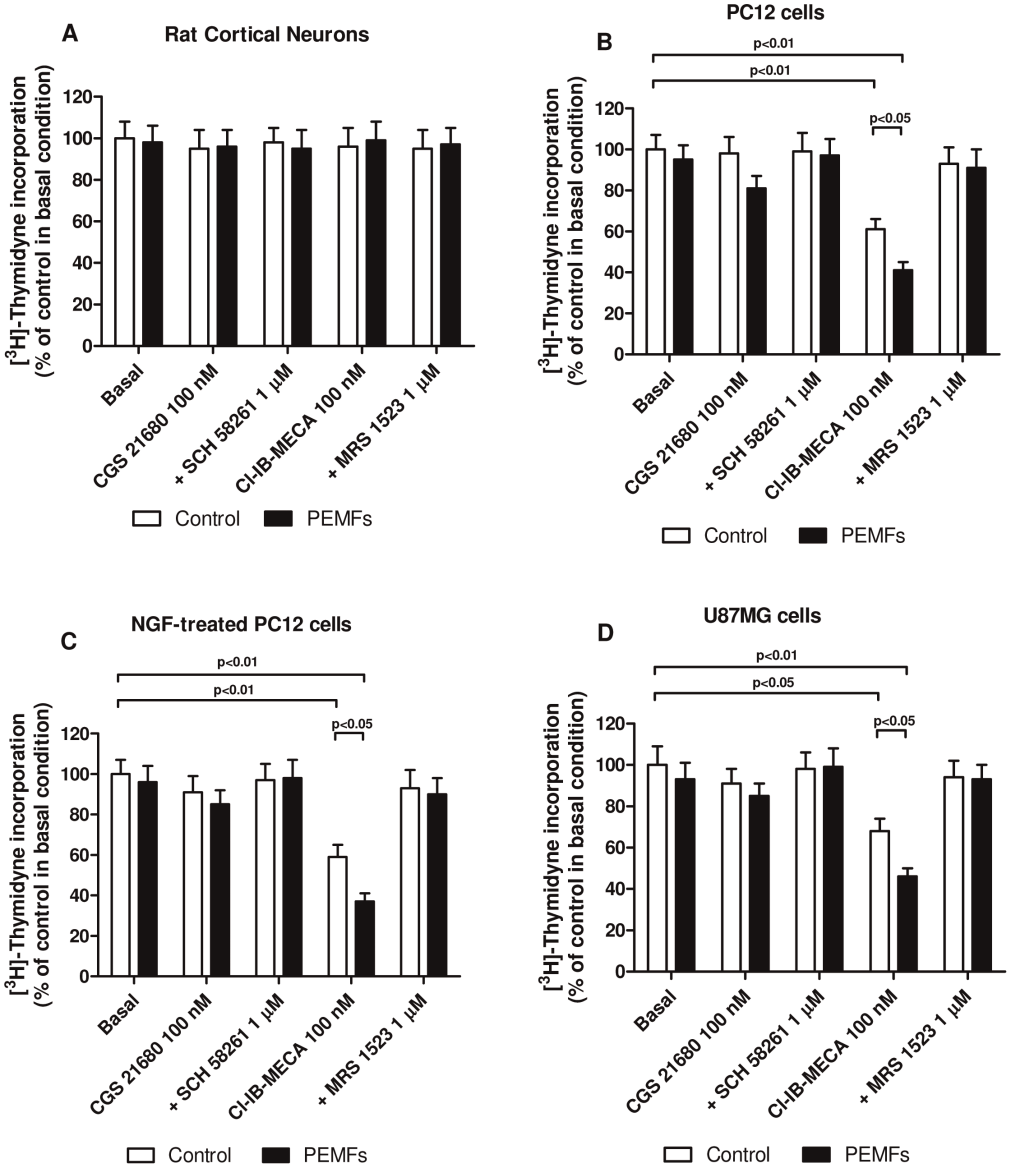
Synergistic effect of A_3_AR stimulation and PEMFs in the inhibition of tumor cell proliferation. Effect of a well-known A_2A_AR agonist and antagonist (CGS 21680, 100 nM; SCH 58261, 1 µM) or A_3_AR agonist and antagonist (Cl-IB-MECA, 100 nM; MRS 1523, 1 µM) in rat cortical neurons, untreated or NGF-treated PC12 cells and U87MG cells in the absence or in the presence of PEMFs on thymidine incorporation. Values are the mean (n = 6) ± SEM.

To evaluate if the modulation of NF-kB and p53 was related to regulation of tumor cell proliferation, a thymidine incorporation assay was performed in the presence or in the absence of PEMFs. In our experimental conditions the basal levels of [^3^H]-thymidine incorporation in rat cortical neurons, untreated or NGF-treated PC12 and U87MG cells were 447±42, 8228±782, 856±78, 9535±844 cpm per sample, respectively. The results indicated that neither CGS 21680 nor PEMFs (alone or in combination) were able to significantly modulate cell proliferation (Fig. 5). The stimulation of A_3_ARs by means of Cl-IB-MECA (100 nM) mediated a significant inhibition of thymidine incorporation of 39%, 41% and 32% in untreated, NGF-treated PC12 cells and U87MG cells, respectively. Notably, PEMF exposure potentiated in a statistically significant way (p<0.05) the effect of Cl-IB-MECA on tumor cell proliferation (Fig. 5B–D). Moreover, A_3_AR stimulation, in the absence or in the presence of PEMFs, did not affect thymidine incorporation in rat cortical neurons, suggesting that the observed effect was selective for tumor cells (Fig. 5). The direct role of A_3_ARs in mediating the effect of Cl-IB-MECA was demonstrated by using a selective A_3_AR antagonist, MRS 1523, that completely abrogated the agonist-induced regulation of p53 and cell proliferation (Figs. 4 and 5).

### PEMF Exposure Enhanced the Cl-IB-MECA-induced Cell Death and Apoptosis in Tumor Cells

**Figure 6:**
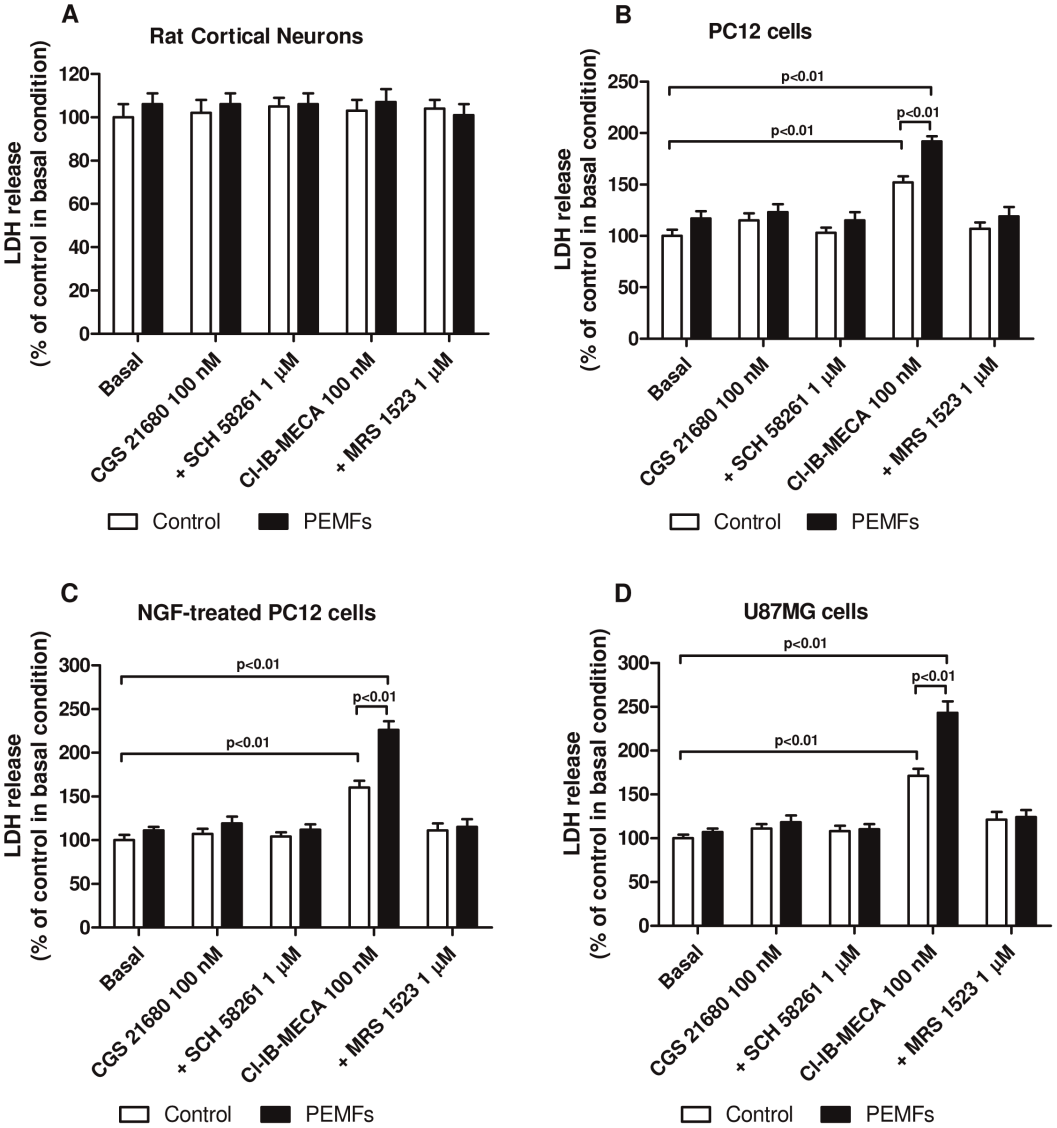
Cytotoxicity of A_3_AR activation and PEMFs in tumor cells. Effect of a well-known A_2A_AR agonist and antagonist (CGS 21680, 100 nM; SCH 58261, 1 µM) or A_3_AR agonist and antagonist (Cl-IB-MECA, 100 nM; MRS 1523, 1 µM) in rat cortical neurons, untreated or NGF-treated PC12 cells and U87MG cells in the absence or in the presence of PEMFs on lactate dehydrogenase (LDH) levels. Values are the mean (n = 6) ± SEM.

**Figure 7:**
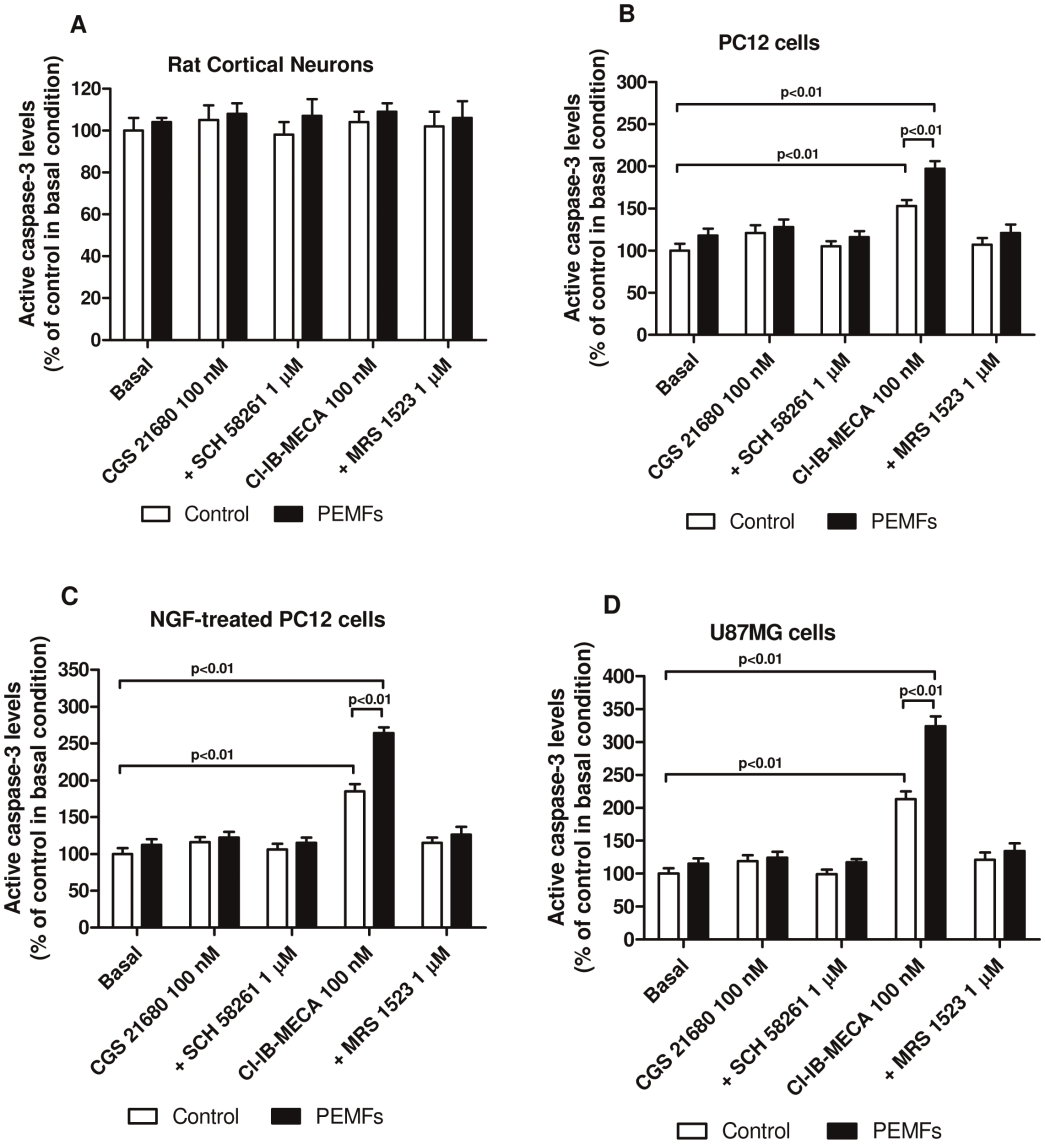
PEMF exposure enhanced the A_3_AR agonist-induced apoptosis in tumor cells. Effect of a well-known A_2A_AR agonist and antagonist (CGS 21680, 100 nM; SCH 58261, 1 µM) or A_3_AR agonist and antagonist (Cl-IB-MECA, 100 nM; MRS 1523, 1 µM) in rat cortical neurons, untreated or NGF-treated PC12 cells and U87MG cells in the absence or in the presence of PEMFs on active caspase 3 levels. Values are the mean (n = 6) ± SEM.

Figure 6 shows the effect of A_2A_ or A_3_AR stimulation on lactate dehydrogenase (LDH) release (% of control in basal condition) from rat cortical neurons, untreated or NFG-treated PC12 cells and U87MG cells in the absence or in the presence of PEMF exposure (1.5 mT). In all the examined cells, the presence of CGS 21680 (100 nM) was not able to modify the LDH release neither in the absence nor in the presence of PEMFs. In rat cortical neurons and tumor cells, PEMF exposure in basal condition was not able to modulate LDH release. The A_3_AR agonist Cl-IB-MECA (100 nM) mediated a cytotoxic effect in all the tumor cells examined as demonstrated by the significant increase of LDH release. Interestingly, the simultaneous presence of PEMFs resulted in a further increase of LDH production mediated by Cl-IB-MECA reaching values of 192%, 226% and 243% respect to the basal condition in untreated, NGF-treated PC12 and U87MG cells, respectively (Fig. 6B–D). To evaluate if the synergistic effect of Cl-IB-MECA and PEMFs involved apoptotic signals, the levels of active caspase-3 were evaluated. Among the different treatment conditions, only the A_3_AR stimulation was able to significantly increase the levels of active caspase-3 in all the tumor cells, but not in rat control cortical neurons (Fig. 7). Furthermore, PEMF exposure enhanced the pro-apoptotic effect of Cl-IB-MECA in cancer cells obtaining an increase of 1.97, 2.64 and 3.24 fold respect to basal condition in untreated or NGF-treated PC12 and U87MG cells, respectively (Fig. 7B–D). The use of a well known A_3_AR antagonist, MRS 1523, reverted the effect of the agonist, suggesting a specific involvement of this receptor subtype.

## Discussion

Numerous studies have demonstrated the involvement of A_3_ARs in the regulation of cell cycle, in pro- and anti-apoptotic effects, in the reduction of cell viability and in the inhibition of cancer growth [4], [15]–[18]. In addition, preclinical and Phase I studies showed that A_3_AR agonists are safe and well tolerated in humans and thus may be considered possible therapeutic agents for certain cancer diseases [5]. As reported in different works some researchers associate EMF exposure with carcinogenesis [30], [31], while other studies have shown that EMF do not increase the risk of several cancer types, and that the treatment with specific frequencies is well tolerated and may have biological efficacy in patients with advanced tumors [32]–[34]. At present, no data are reported in literature on the effect of ARs and PEMF exposure in *in vitro* or *in vivo* models of cancer, even if some results have demonstrated the potentiation of the anti-inflammatory effects of ARs by the presence of PEMFs [22]–[25].

In this paper we have combined the role of A_3_ARs as a target for cancer with the growing observation that PEMFs may have beneficial effects in the modulation of physiological processes associated with tumor cell signaling. More specifically the current experiments demonstrate that PEMFs modulate the expression and the effect of A_3_ARs in different cultured neural tumor cells represented by PC12 and U87MG cells in comparison with rat cortical neurons. It is well reported that cell lines are useful in pharmacological studies because they offer an opportunity to study signal transduction and regulation of receptors under more controlled conditions than in native cells or tissues [41]. Moreover, we have treated PC 12 cells with NGF to evaluate the functional responses of tumor cells differentiated in neuron-like cells. Using these cellular models we highlighted the combined effect of PEMF exposure and A_3_AR stimulation in different physiological responses such as NF-kB and p53 activation, cell proliferation, cytotoxicity and apoptosis.

First of all, PEMF exposure significantly increased the A_2A_ and A_3_AR mRNA and density in tumor cells as well as in rat cortical neurons. Saturation binding experiments of A_2A_ and A_3_ARs performed after PEMF exposure in rat cortical neurons, in untreated or NGF-treated PC12 cells and in U87MG cells (membranes or intact cells) revealed a significant increase in Bmax values in comparison with unexposed cells suggesting the modulation of PEMF treatment on the receptor density. These data are well correlated with our previous results showing that PEMFs are able to up-regulate A_2A_ and A_3_AR density and functionality in human neutrophils, human and bovine synoviocytes, and bovine chondrocytes [22]–[25]. Moreover these results are closely associated with recent data showing the increase of A_2A_ARs in rat cortical neurons following PEMF exposure [42]. At the present, the mechanisms of PEMF action are still poorly defined even if at a molecular level could involve modifications of receptor recycling to the cell membrane as well as a regulatory effect at a transcriptional level, as suggested by the increase of A_2A_ and A_3_AR mRNA following PEMF exposure.

To investigate if the up-regulation of A_2A_ and A_3_ARs was associated with a high functionality of these receptors, different cellular responses have been studied. First of all we have evaluated the effect of well known adenosine agonists such as CGS 21680 and Cl-IB-MECA on cAMP accumulation. In the cell lines investigated the stimulation of CGS 21680 on A_2A_ARs was increased after the treatment with PEMFs. Similarly, the inhibitory effect of Cl-IB-MECA was potentiated by PEMF exposure in primary rat cortical neurons, untreated or NGF-treated PC12 cells and U87MG cells suggesting that the increase of the receptors is associated with an increase of receptor functionality. Because of the close association between NF-kB signal transduction pathways and a wide range of cellular processes including proliferation, survival and apoptosis we analyzed the role of A_2A_ and A_3_ARs in the absence and in the presence of PEMFs. We found that, in tumor cells, PEMF exposure was able to enhance the reduction of NF-kB induced by A_2A_ and A_3_AR stimulation. Since the crosstalk between NF-kB and p53 oncosuppressor protein is recognized to have a crucial role in the development of cancer [43], we have evaluated the effect of A_2A_ and A_3_AR activation and PEMFs exposure on p53 protein levels. In tumor cells, but not in rat cortical neurons, the A_3_AR agonist Cl-IB-MECA mediated an increase of p53 levels, an effect that was enhanced by the presence of PEMFs. These results are in agreement with those found in a recent work, showing that an A_3_AR agonist inhibited prostate cancer cells proliferation and induced G1 cell cycle arrest through the p53 induction [44]. To evaluate if the regulation of these fundamental factors was related with the modulation of tumor cell proliferation, a thymidine incorporation assay was performed. An inhibitory effect of Cl-IB-MECA on cell proliferation was found in tumor cells confirming the involvement of A_3_AR activation in blocking tumor development and was much more evident in the presence of PEMF exposure. This anti-proliferative effect was potentiated by PEMFs in the tumor cells examined. The PEMF treatment did not modify the proliferation in cortical neurons in the absence or in the presence of AR stimulation even if the low proliferative index could represent a drawback. In addition, these effects were abrogated by the presence of A_3_AR antagonist suggesting the direct involvement of this specific AR subtype. Nowadays, the anti-proliferative effect of Cl-IB-MECA has been extensively investigated with conflicting results due to the tissues or cells analyzed. For example it has been demonstrated that Cl-IB-MECA derivatives mediate a reduction of cell proliferation through cell cycle arrest in human lung cancer cells suggesting the primary role of A_3_ARs to inhibit tumor cell proliferation and cell migration [45]. We have also found that the A_3_AR agonist Cl-IB-MECA mediated a cytotoxic effect in the different tumor cells examined as demonstrated by the significant increase of LDH release. The effect of A_3_AR stimulation on LDH release was potentiated by PEMF exposure in untreated or NGF treated PC12 cells and in U87MG cells. Notably, no cytotoxic effect of Cl-IB-MECA was found in rat cortical neurons, suggesting that this effect could be associated to the tumor cells. These results are in agreement with those previously reported demonstrating that A_3_AR agonists potentiate the cytotoxic effect of the chemotherapeutic agents in colon carcinoma cells [46]. In order to evaluate if the observed effect of A_3_AR activation on tumor cell signaling and death also involved apoptotic events, active caspase-3 levels were investigated following the treatment with AR agonists in the presence or in the absence of PEMF exposure. Neither CGS 21680 nor Cl-IB-MECA, alone or in combination with PEMFs, were able to affect active caspase-3 levels in rat cortical neurons. Interestingly, in tumor cells, Cl-IB-MECA, but not CGS 21680 was able to increase caspase-3 levels, an effect that was enhanced by PEMF exposure, suggesting the synergistic role of A_3_AR stimulation and PEMFs in the induction of apoptosis. These results are consistent with those recently reported showing that the A_3_AR agonist IB-MECA induced apoptosis in rat prostate cancer cell lines and in the human metastatic androgen-independent prostate cancer cell line [18]. All these data confirm previous papers where in different tumors it has been found a direct involvement of A_3_AR expression and functionality [14], [47].

Despite the increasing amount of experimental data on PEMFs, controversial effects have been reported probably due to the various systems and apparatus used in *in vivo* or *in vitro* experiments. In particular, the different physical variables such as frequency, intensity and the waveform could be used on empirical basis. In this study, we have used a well characterized PEMF exposure system, based on rational driven methodology, which was able to modulate in various non-tumor cell types, the expression and functionality of ARs as previously reported [22]–[25], [42], [48]. *In vitro* experiments on different tumor cell lines have suggested that PEMF application combined with anticancer drugs will be very effective as non invasive applications to tumor therapy [49]. In an *in vivo* animal model the application of low frequency EMF inhibits preneoplastic lesions chemically induced in the rat liver through the reduction of cell proliferation [50]. Recently, in clinical studies with various types of cancers by using a non-invasive feedback method to identify tumor-specific frequencies and to test the feasibility of administering such frequencies to patients with advanced cancer were performed and may lead to the discovery of novel pathways controlling cancer growth [34].

Previous *in vitro* assays have reported that A_3_AR agonists exert a differential effect on normal and tumor cells. In normal cells, the agonists induce the production of growth factors and in tumor cells, the agonists induce apoptosis and tumor growth inhibition via deregulation of the NF-kB and the Wnt signaling pathways [5]–[7], [11], [12]. Our results are in agreement with these data and confirm the dual role of A_3_ARs raising the question about the mechanism of this differential effect. A possible explanation could be related to the different expression of crucial regulatory factor in tumor cells. It is well known that NF-kB is highly expressed in tumor cells where its constitutive activation appears to affect cancer cell survival by promoting anti-apoptotic genes expression [51], [52]. In our study, the low concentration of Cl-IB-MECA was most likely sufficient to inhibit NF-kB in tumor cells without affecting this pathway in control cells. Analogously, the effect of A_3_AR stimulation on dysregulated p53 protein in tumor cells could be on the basis of the different responses observed respect to the control cells. PEMF exposure potentiated these effects in tumor cells without increase the concentration of the A_3_AR agonist. From the pharmacological point of view, a possible advantage of the combination of low dose agonist and of a localized external PEMF therapy could be the reduction of the risk of adverse or systemic side effects that increase dramatically when the drugs are administered at high dose.

These results indicate the possibility that the anti-tumor effect mediated by A_3_ARs could be potentiated by a non-invasive stimulus represented by PEMFs, allowing to use of low concentrations of the agonist limiting its potential side effects. The pro-apoptotic and anti-proliferative effect promoted by the A_3_AR agonist Cl-IB-MECA seems to be specific for tumor cells without affecting rat primary neurons. Future *in vivo* studies are therefore warranted to better investigate the synergistic interaction between A_3_ARs and PEMF exposure and to further confirm the efficacy of this treatment as well as to evaluate adverse effects.

## Materials and Methods

### Preparation of Rat Brain Cortical Neurons, PC12 and U87MG Cells

Primary rat cortical cells isolated from day-18 rat embryos containing minimum amount of astrocytes and other glial cells were purchased from Invitrogen (Carlsbad, CA, USA). These highly pure cells were grown in Neurobasal Medium containing B27 supplements (Invitrogen). Before using, cells were washed twice with serum-free medium and treatments were carried out in Neurobasal medium without phenol red and B27 supplements without anti-oxidants [53].

Rat pheochromocytoma (PC12) cells were purchased from American Type Culture Collection (Manassas, VA, USA) and were maintained in DMEM medium supplemented with 5% FBS, 10% horse serum, L-glutamine (2 mM), penicillin (100 U/ml) and streptomycin (100 µg/ml) in a humidified atmosphere (5% CO_2_) at 37°C. Cells were subcultured three times a week at a density of 500000/ml. Differentiation was achieved by treatment with 50 ng/ml nerve growth factor (NGF, Sigma, St Louis, MO) for 1 week [54].

U87MG (malignant glioma) cells were purchased from American Type Culture Collection (Manassas, VA, USA). U87MG cells were maintained in Dulbecco's modified Eagle's medium/Ham's F12 medium (DMEM/F12 medium) with 20% fetal calf serum, 2 mM l-glutamine, 100 U/ml penicillin, 100 µg/ml streptomycin in a humidified atmosphere (5% CO_2_) at 37°C [55].

### Electromagnetic Field Exposure System

Rat cortical neurons, untreated or NGF-treated PC12 and U87MG cells were exposed to PEMFs generated by a pair of rectangular horizontal coils (14 cm × 23 cm), each made of 1400 turns of copper wire placed opposite to each other. The culture was placed between this pair of coils so that the plane of the coils was perpendicular to the culture flasks. The coils were powered by the PEMF generator system (IGEA, Carpi, Italy) used in previous studies [22]–[25], which produced a pulsed signal with the following parameters: pulse duration of 1.3 ms and frequency of 75 Hz, yielding a 0.1 duty cycle. The peak intensity of the magnetic field and peak intensity of the induced electric voltage were detected in air between two coils from one side to the other, at the level of the culture flasks. The peak values measured between two coils in air had a maximum variation of 1% in the whole area in which the culture flasks were placed. The dimensions of the flasks were 9.2 cm × 8.2 cm with 10 ml of medium. The peak intensity of the magnetic field was 1.5 ± 0.2 mT and it was detected using the Hall probe (HTD61-0608-05-T, F.W. Bell, Sypris Solutions, Louisville, KY) of a gaussmeter (DG500, Laboratorio Elettrofisico, Milan, Italy) with a reading sensitivity of 0.2%. The corresponding peak amplitude of the induced electric voltage was 2.0 ± 0.5 mV. It was detected using a standard coil probe (50 turns, 0.5 cm internal diameter of the coil probe, 0.2 mm copper diameter) and the temporal pattern of the signal was displayed using a digital oscilloscope (Le Croy, Chestnut Ridge, NY). The shape of the induced electric voltage and its impulse length were kept constant.

### Cell Treatment and Preparation

The primary rat cortical cells, PC 12 and U87MG cells were also treated with well known A_2A_ and A_3_AR agonists such as 2-[p-(2-carboxyethyl)-phenethylamino]-5′-N-ethylcarboxamido-adenosine (CGS 21680, 100 nM, Sigma) and 2-chloro-*N* ^6^-(3-iodobenzyl) adenosine-5′-*N*-methyl-uronamide (Cl-IB-MECA, 100 nM, Tocris, Bristol, UK) in the absence or in the presence of the selective A_2A_ and A_3_AR antagonists 5-amino-7-(phenylethyl)-2-(2-furyl)-pyrazolo[4,3-e]-1,2,4-triazolo[1,5-c]pyrimidine (SCH 58261, 1 µM, Tocris) and 3-Propyl-6-ethyl-5-[(ethylthio) carbonyl]-2 phenyl-4-propyl-3-pyridine carboxylate (MRS 1523, 1 µM, Sigma), respectively. The primary rat cortical cells, PC 12 and U87MG cells were then incubated at 37°C for 24 hours in the presence or in the absence of PEMFs before the A_1_, A_2A_, A_2B_ and A_3_AR saturation binding experiments and functional assays. No change of temperature was measured during time of PEMF treatment. Membranes were also prepared from the cells described above and were used to evaluate AR binding parameters. Briefly, the cells were harvested by scraping from the plates, homogenized with a Polytron (Kinematica, Bohemia, NY, USA) and the membranes were then collected by centrifugation at 40000×g for 15 min at 4°C. The protein concentration was determined according to a Bio-Rad method with bovine albumin as reference standard [22]–[24]. Saturation binding experiments were performed by using intact cells or membranes from rat cortical neurons, PC 12 and U87MG cells.

### Saturation Binding Experiments for A_1_, A_2A_, A_2B_ and A_3_ Adenosine Receptors

Saturation binding experiments to A_1_ARs were performed according to the method previously described using [^3^H]-1,3-dipropyl-8-cyclopentyl-xanthine ([^3^H]-DPCPX, specific activity 120 Ci/mmol; Perkin Elmer Life and Analytical Sciences, Boston, MA, USA) as radioligand [56]. The membranes (100 µg of protein/assay) with 8 to 10 concentrations of the radioligand [^3^H]-DPCPX (0.1–10 nM) were incubated in Tris HCl 50 mM, pH 7.4, for 90 min at 4°C. Non specific binding was determined in the presence of DPCPX 1 µM.

Saturation binding experiments to A_2A_ARs were performed by using [^3^H]-4-(2-[7-amino-2-(2-furyl) [1], [2], [4] triazolo[2,3-a] [1], [3], [5] triazin-5-yl- amino]ethyl ([^3^H]-ZM 241385, specific activity 27.4 Ci/mmol; American Radiolabeled Chemicals Inc, Saint Louis, MO, USA) as radioligand [22], [41]. The membranes (100 µg of protein/assay) were incubated for 60 min at 4°C with 8 to 10 concentrations of the radioligand [^3^H]-ZM 241385 (0.1–20 nM) and Tris HCl 50 mM, MgCl_2_ 10 mM, pH 7.4. Non specific binding was determined in the presence of ZM 241385 1 µM.

Saturation binding experiments to A_2B_ARs were analyzed using [^3^H]-MRE 2029F20 ([^3^H]-N-benzo [1], [3]dioxol-5-yl-2-[5-(2,6-dioxo-1,3-dipropyl-2,3,6,7-tetrahydro-1H-purin-8-yl)-1-methy-1H-pyrazol-3-yl-oxy]-acetamide, specific activity 123 Ci/mmol, GE Healthcare, UK) as radioligand [57]. Cell membranes (80 µg of protein/assay) and [^3^H]-MRE 2029F20 (0.1–20 nM) were incubated for 60 min at 4°C and non specific binding was determined in the presence of MRE 2029F20 (1 µM).

Saturation binding experiments to A_3_ARs were assessed using [^125^I]-AB-MECA ([^125^I]-4-aminobenzyl-5′-N-methylcarboxamidoadenosine, specific activity 2000 Ci/mmol, Perkin Elmer) as radioligand [56]. The membranes (50 µg of protein/assay) with [^125^I]-AB-MECA (0.1-10 nM) were incubated at 37°C for 60 min and R-PIA (R-N^6^-phenylisopropyl adenosine) 50 µM was used to evaluate non specific binding.

Saturation binding experiments were also performed in intact cells derived from the cell lines examined (10^6^ cells/sample) with an incubation time of 120 min for A_1_, A_2A_ and A_2B_ ARs and 60 min for A_3_ARs at the temperature of 37°C.

Bound and free radioactivity were separated by filtering the assay mixture through Whatman GF/B glass fibre filters by use of a Brandel cell harvester (Brandel Instruments, USA). The filter bound radioactivity was counted by Scintillation Counter Packard Tri Carb 2810 TR (Perkin Elmer, USA).

### Real-Time Quantitative Polymerase Chain Reaction (RT-PCR) Experiments

Total cytoplasmic RNA was extracted from primary rat cortical cells, PC12 and U87MG cells by the acid guanidinium thiocyanate phenol method. Quantitative RT-PCR assays of A_1_, A_2A,_ A_2B_ and A_3_AR mRNAs were carried out using gene-specific fluorescently labelled TaqMan MGB probe (minor groove binder) in a ABI Prism 7700 Sequence Detection System (Applied Biosystems, Warrington Cheshire, UK [58]. For the RT-PCR of A_1_, A_2A,_ A_2B_ and A_3_ARs the assays-on-demand™ Gene expression Products NM 000674, NM 000675, NM 000676 and NM 000677 were used respectively. For the RT-PCR of the reference gene, the endogenous control human β-actin kits was used, and the probe was fluorescent-labeled with VIC™ (Applied Biosystems, Monza, Italy).

### cAMP Production Assays

The rat cortical cells, PC12 and U87MG cells (10^6^ cells per sample) were suspended in 0.5 ml incubation mixture Krebs Ringer phosphate buffer, containing 1.0 IU/ml adenosine deaminase (Sigma) and preincubated for 10 min in a shaking bath at 37°C. To better investigate the inhibitory effect of Cl-IB-MECA, the cells were also incubated with forskolin (1 µM) and/or 0.5 mM of 4-(3-butoxy-4-methoxybenzyl)-2-imidazolidinone (Ro 20-1724) as phosphodiesterase inhibitor. The effect of CGS 21680 or Cl-IB-MECA was also evaluated in the presence of selected antagonists such as SCH 58261 or MRS 1523 at 1 µM concentration. The final aqueous solution was tested to evaluate cAMP levels by using a competition protein binding assay with [^3^H]-cAMP, trizma base 100 mM, aminophylline 8.0 mM, mercaptoethanol 6.0 mM, pH 7.4 [24]. At the end of the incubation time (150 min at 4°C) and after the addition of charchoal the samples were centrifuged at 2000×g for 10 min and the clear supernatant was counted in a liquid Scintillation Counter Tri Carb Packard 2810 TR (Perkin-Elmer).

### NF-kB Activation Assays

Nuclear extracts from the examined cells (primary rat cortical cells, PC12 and U87MG cells) were obtained by using a nuclear extract kit (Active Motif, Carlsbad, USA) according to the manufacturer instructions. The NF-kB activation was evaluated by detecting phosphorylated p65 proteins in nuclear extracts by using the TransAM NF-kB kit (Active Motif). Phosphorylated NF-kB subunits specifically binds to the immobilized oligonucleotides containing the NF-kB consensus site (5′-GGGACTTTCC-3′). The primary antibody used to detect NF-kB recognized an epitope in the subunits that is accessible only when it is activated and bound to its DNA target. A horseradish peroxidase (HRP)-conjugated secondary antibody provided a sensitive colorimetric readout that was quantified by spectrophotometry at 450 nm wavelength [58].

### p53 ELISA Assay

For the detection of p53, the pan p53 ELISA kit (Roche Molecular Biochemicals, Germany) was used in primary rat cortical cells, PC12 and U87MG cells. Briefly, cell lysate and biotinylated mouse monoclonal antibody were placed in 96 well microtiter plates coated with mouse monoclonal antibody specific for human p53, and were incubated for 2 hours at room temperature. After removing unbound material by washing with PBS, horseradish peroxidase conjugated streptavidin was added to bind to the antibodies. The absorbance in each well was measured at 450 nm, and concentrations of p53 were determinate by interpolating from standard curves obtained with known concentrations of p53 protein [59].

### Cell Proliferation Assays

All the cells examined, in the different conditions described above, were seeded (10^5^/cells per well) in fresh medium with 1 µCi/ml [^3^H]-Thymidine for 24 hours in Dulbecco’s modified Eagle’s medium containing 10% fetal calf serum, penicillin (100 units/ml), streptomycin (100 µg/ml). After 24 hours of labeling, cells were trypsinized, dispensed in four wells of a 96-well plate, and filtered through Whatman GF/C glass fiber filters using a Micro-Mate 196 cell harvester (Perkin Elmer). The filter bound radioactivity was counted on Top Count Microplate Scintillation Counter with Micro Scint 20 [60].

### Lactate Dehydrogenase Assay

LDH is a soluble cytosolic enzyme that is released into the culture medium following loss of membrane integrity resulting from either apoptosis or necrosis. LDH activity can be used as an indicator of cell membrane integrity and serves as general meaning to assess cytotoxicity resulting from chemical compounds or environmental toxic factors [53].

LDH activity present in the culture medium was evaluated using a coupled two-step reaction. In the first step, LDH catalyzes the reduction of NAD^+^ to NADH and H^+^ by oxidation of lactate to pyruvate. In the second step of the reaction, diaphorase uses the newly-formed NADH and H^+^ to catalyze the reduction of a tetrazolium salt to highly-colored formazan which absorbs strongly at 490–520 nm.

After 24 hours following treatment of the cells (primary rat cortical cells, PC12 and U87MG cells), 100 µl of supernatant per well was harvested and transferred into a new 96-well, flat-bottom plate. LDH substrate (100 µl) was added to each well and incubated for 30 min at room temperature protected from light. The absorbance of the samples was measured at 490 mm on spectrophotomer [58].

### Apoptosis Assay

Apoptosis assay was performed evaluating active caspase-3 levels after the treatment of the cells with A_2A_ or A_3_AR ligands and/or PEMF exposure. After 24 hours, the cells were treated with biotin-ZVKD-fmk inhibitor (10 µM) for one hour at room temperature. After discarding the culture media, cells were rinsed with PBS and the extraction buffer containing protease inhibitors was added to prepare cell extracts. After two hours of incubation at room temperature 100 µl of samples were transferred into a microplate pre-coated with a monoclonal antibody specific for caspase-3. After washing, 100 µl of streptavidin caspase-3 conjugated to horseradish peroxidases that binds to the biotin of the inhibitor were added. Following the wash, the substrate solution was added to the wells for 30 min and stop solution was used to block the reaction. The optical density was determined using a microplate reader set to 450 nm [58].

### Statistical Analysis

Dissociation equilibrium constants for saturation binding, affinity or K_D_ values, as well as the maximum densities of specific binding sites, Bmax, were calculated for a system of one or two-binding site populations by non-linear curve fitting using the program Ligand (Kell Biosoft, Ferguson, MO) [58]. Analysis of data was performed by one-way analysis of variance. Differences between the groups were analyzed with the unpaired t-test and were considered significant at a value of p<0.05. All experimental data are reported as mean±SEM of 6 independent experiments.
